# An optogenetic system for interrogating the temporal dynamics of Akt

**DOI:** 10.1038/srep14589

**Published:** 2015-10-01

**Authors:** Yoshihiro Katsura, Hiroyuki Kubota, Katsuyuki Kunida, Akira Kanno, Shinya Kuroda, Takeaki Ozawa

**Affiliations:** 1 Department of Chemistry, School of Science, The University of Tokyo, 7-3-1 Bunkyo-ku, Hongo, Tokyo 113-0033, Japan; 2 Department of Biological Sciences, School of Science, The University of Tokyo, 7-3-1 Bunkyo-ku, Hongo, Tokyo 113-0033, Japan; 3 Division of integrated Omics, Research Center for Transomics Medicine, Medical Institute of Bioregulation, Kyushu University, 3-1-1, Maidashi, Higashi-ku, Fukuoka, Fukuoka 812-8582, Japan; 4 CREST, Japan Science and Technology Agency, 4-1-8 Honcho, Kawaguchi, Saitama, 332-0012, Japan

## Abstract

The dynamic activity of the serine/threonine kinase Akt is crucial for the regulation of diverse cellular functions, but the precise spatiotemporal control of its activity remains a critical issue. Herein, we present a photo-activatable Akt (PA-Akt) system based on a light-inducible protein interaction module of *Arabidopsis thaliana* cryptochrome2 (CRY2) and CIB1. Akt fused to CRY2phr, which is a minimal light sensitive domain of CRY2 (CRY2-Akt), is reversibly activated by light illumination in several minutes within a physiological dynamic range and specifically regulates downstream molecules and inducible biological functions. We have generated a computational model of CRY2-Akt activation that allows us to use PA-Akt to control the activity quantitatively. The system provides evidence that the temporal patterns of Akt activity are crucial for generating one of the downstream functions of the Akt-FoxO pathway; the expression of a key gene involved in muscle atrophy (*Atrogin-1*). The use of an optical module with computational modeling represents a general framework for interrogating the temporal dynamics of biomolecules by predictive manipulation of optogenetic modules.

The dynamic spatiotemporal balance of various signaling pathways is crucial for living organisms^1^,^2^,^3^. The Akt (also known as protein kinase B) signaling pathway plays a key role in a variety of biological functions^4^. With advances in systems biology and imaging technologies such as fluorescent resonance energy transfer (FRET) biosensors, the significance of spatiotemporal Akt activity has become of great interest^5^,^6^,^7^. The temporal patterns of Akt activity have been suggested to determine the cellular outcome of insulin stimulation^5^. Additionally, specific spatial patterns of Akt activity have been observed following stimulation with specific extracellular ligands^6^,^7^. Furthermore, disruption of these patterns is often associated with human diseases; for example, abnormal patterns of Akt, such as hyper-activation and desensitization cause numerous diseases including tumorigenesis and type II diabetes^8^,^9^. However, it is currently unclear how and to what extent patterns of Akt activity contribute to the functions of whole living systems, because no methods exist that can reconstruct physiological spatiotemporal patterns of Akt activity. Therefore, the ability to control Akt activity with spatiotemporal precision is crucial for elucidating its complicated physiological functions.

Optogenetic manipulation of ion channels has provided unique approaches for studying neuronal systems^10^. Additionally, techniques such as the optical regulation of small-GTPase activity, ERK activity, epigenetic states, and phosphoinositide metabolism have enabled researchers to elucidate fundamental cellular functions in living systems^11^,^12^,^13^,^14^,^15^. An important challenge for controlling optogenetic tools is the predictive and quantitative control of the light-induced output. One method to address this issue is the use of an on-line feedback system in which the optogenetic output is monitored with fluorescence imaging^16^,^17^. However, this method lacks generality owing to the limitation of selecting fluorescent dyes and proteins because optogenetic systems preclude the use of fluorophores with absorption spectra that overlap the spectra of the optogenetic modules.

In the present study, we describe a method for specifically controlling Akt activity in living cells with external light stimulation. Computational modeling of the optogenetic output enables quantitative control of temporal Akt activity and allows the interrogation of the functional significance of its temporal patterns.

## Results

### Development of an optogenetic Akt system

**Figure 1:**
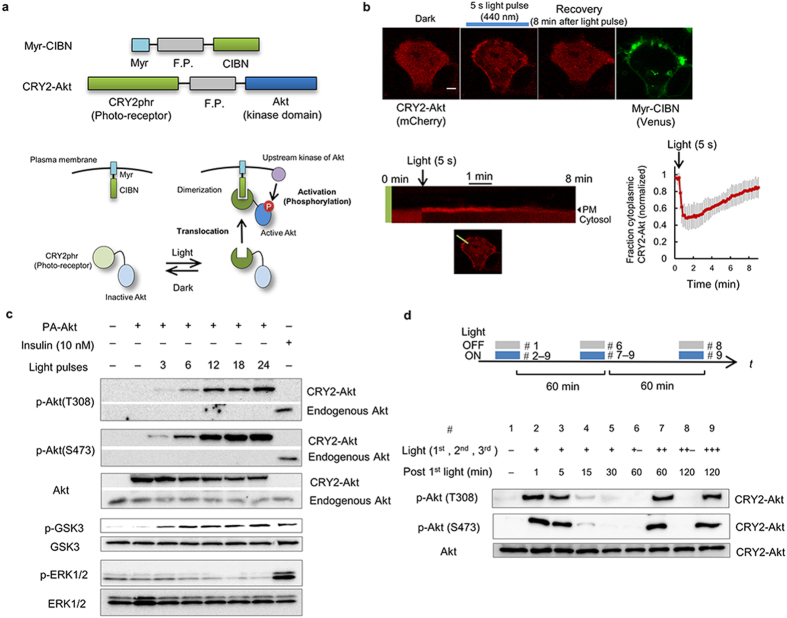
Photo-activatable Akt (PA-Akt) system. (**a**) Schematic of a PA-Akt system. Upon light stimulation, inactive CRY2-Akt in cytosol is translocated to the plasma membrane and is subsequently activated by upstream kinases. CRY2–CIBN dimer dissociates under a dark condition, enabling reversible spatiotemporal control of Akt activity. Fusion proteins were labeled with a fluorescent protein (F.P.). (**b**) Light-induced membrane localization of CRY2-Akt in HEK293 cells. The cell expressing CRY2-Akt and Myr-CIBN was stimulated with 440-nm laser light for 5 s. A kymograph shows the change of fluorescence intensity of CRY2-Akt over 8 min along the green line in the left image. Green, Myr-CIBN; Red, CRY2-Akt; Scale bar, 5 μm. The graph shows the time course of normalized fluorescence intensity of CRY2-Akt at cytoplasm. Bars: Mean ± S.D. (*N* = 19). (**c**) Dose-dependent activation of Akt signaling with light. C2C12 cells expressing Myr-CIBN and CRY2-Akt were stimulated with different times of light pulses at 1-min interval. One minute later after the final light pulse, the cells were collected and subjected to Western blot assay. Insulin was added for 15 min. **(d)** Reversibility of CRY2-Akt activation. CRY2-Akt was activated three times each at an interval of 60 min. In each activation, cells were stimulated 12 times with 1-min interval of light pulses at an intensity of 4 mW/cm^2^.

The kinase activity of Akt is regulated by its plasma membrane localization and subsequent phosphorylation by upstream kinases. Upon external stimulation by factors such as growth factors and hormones, phosphoinositide 3-kinase (PI3K) synthesizes the lipid molecule phosphatidylinositol (3,4,5)-trisphosphate (PIP3), which recruits Akt to the plasma membrane. Membrane-localized Akt is activated by phosphorylation at two sites, Thr308 by PDK1 and Ser473 by mTORC2. To generate photo-activatable Akt (PA-Akt), we tested three light-inducible systems using a CRY2–CIB1 dimerization system (Fig. 1a and Supplementary Fig. 1)^18^. Based on the criterion that the system must enable control of Akt localization reversibly with minimal domains, we chose a system in which the kinase domain of Akt was fused with CRY2phr, a minimal light sensitive domain of CRY2 (named CRY2-Akt) and the N-terminal portion of CIB1 (CIBN) was fused with a membrane-targeting myristoylation sequence (named Myr-CIBN) (Fig. 1a). Only the kinase domain of Akt was fused with CRY2phr to minimize the activation of the optogenetic module without light because full-length Akt contains the PH domain responsible for PIP3 binding. Under dark conditions, Myr-CIBN (labeled with the yellow fluorescent protein Venus) was localized at the plasma membrane of HEK293 cells; in contrast, CRY2-Akt (labeled with the red fluorescent protein mCherry) was localized in the cytosol. CRY2-Akt was reversibly translocated to the plasma membrane upon stimulation of 440-nm light. The kinetics were similar to those previously reported for CRY2−CIB systems^18^ (Fig. 1b and Supplementary Video 1). The localization was regulated with subcellular resolution in the cell (Supplementary Fig. 1d and Supplementary Video 2).

To evaluate the activation of membrane-localized CRY2-Akt, we investigated its phosphorylation at Thr308 and Ser473 in C2C12 cells expressing Myr-CIBN and CRY2-Akt. The phosphorylation level increased directly with repeated pulses of 470-nm light and reached a plateau following more than 12 pulses (Fig. 1c). The dynamic range of the phosphorylation was set to the comparable level to those of endogenous Akt and constitutively membrane-localized Akt (Myr-Akt) (Supplementary Fig. 2). The light intensity and duration were optimized to rapidly activate CRY2-Akt (Supplementary Fig. 3). Under each fixed condition of light intensity and duration, CRY2-Akt was phosphorylated in proportion to the number of light pulses. Termination of the light stimulation caused CRY2-Akt inactivation within tens of minutes and further light stimulation caused repeated activation (Fig. 1d). The kinase activity of the phosphorylated CRY2-Akt was confirmed by the phosphorylation of the Akt substrate, GSK3 (Fig. 1c). Importantly, unlike insulin stimulation, which simultaneously activates several pathways including Akt and ERK, light stimulation achieved specific activation of Akt signaling (see the p-ERK1/2 column in Fig. 1c). Interestingly, specific Akt activation resulted in the reduced phosphorylation of ERK, which is consistent with previous reports^19^.

### Optical control of the downstream functions of Akt

**Figure 2:**
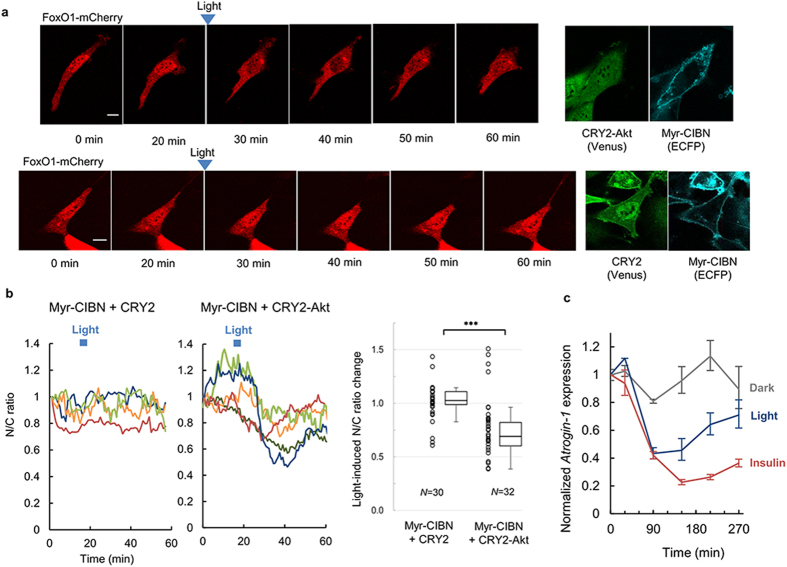
Optical control of Akt-FoxO pathway. (**a**) Time-lapse images of FoxO1 upon CRY2-Akt activation in C2C12 cells. Myr-CIBN was labeled with ECFP, and CRY2-Akt with Venus. Scale bars, 10 μm. (**b**) (Left) Representative time courses of Nuclear/Cytoplasm (N/C) fluorescence ratio of FoxO1-mCherry. (Right) Box and whisker plot of the N/C ratio change upon light illumination with outliers. N/C ratio (before light) was obtained by the average of 5 images taken during 15 min prior to light illumination, while N/C ratio (after light) was obtained by the average of 5 images during 15 min, which were taken from 10 min later post light illumination. (*N* = 30 for control cells expressing Myr-CIBN and CRY2, *N* = 32 for cells expressing Myr-CIBN and CRY2-Akt, ***p < 0.001 by a two-tailed Student’s *t*-test). **(c)** Light-induced down-regulation of FoxO1-regulated gene expression. Cells expressing Myr-CIBN and CRY2-Akt were continuously stimulated with 1-min interval of light pulses at 1 mW/cm^2^ intensity or 10 nM insulin. Bars: Mean ± s.e.m. (*N* = 4).

To further characterize the present system, we examined the perturbation of downstream biological processes of Akt. The transcriptional factor FoxO1 is a key regulator of cell metabolism, and its subcellular localization is controlled by Akt-mediated phosphorylation^20^. FoxO1 (labeled with mCherry) was co-expressed with Myr-CIBN (labeled with the enhanced cyan fluorescent protein ECFP) and CRY2-Akt (labeled with Venus) in C2C12 cells. Nuclear-localized FoxO1-mCherry was translocated to the cytosol upon light illumination (Fig. 2a,b). Interestingly, Akt activation caused oscillations in the nuclear abundance of FoxO1-mCherry in 5 of the 32 analyzed cells (Supplementary Video 3). Additionally, the expression of the FoxO1-regulated gene encoding muscle-specific ubiquitin ligase, *Atrogin-1*^21^, decreased 90 min after the onset of light illumination, but the amplitude of the decrease at the later time points was smaller in light-stimulated cells than in cells stimulated with insulin (Fig. 2c). These results underscore the central role of Akt in regulating FoxO1 activity compared with other factors, such as FoxO1 acetylation, ubiquitination, and phosphorylation by other kinases^22^, which would be necessary for a full decrease in *Atrogin-1* expression. In addition, we confirmed that the expression of Myr-CIBN and CRY2-Akt itself did not significantly alter the basal level of *Atrogin-1* expression, whereas the basal localization of FoxO1-mCherry was slightly affected by the expression of those constructs. The results indicate that the over-expressed CRY2-Akt with Myr-CIBN might have a quite weak activity, causing nuclear export of the FoxO1-mCherry to a small extent (Supplementary Fig. 4a,b). Furthermore, we investigated the effect of the activation of Akt signaling on actin remodeling. The results of previous studies suggested that Akt activity was involved in actin reorganization, although the detailed mechanisms remain unknown^23^,^24^. We found that membrane ruffling formation occurred in C2C12 cells upon CRY2-Akt activation (Supplementary Fig. 5a and Supplementary Video 4). Moreover, focal light stimulation perturbed cell polarity (Supplementary Fig. 5b,c and Supplementary Video 5), which indicated that Akt activity was a driving force in actin reorganization. Taken together, these results demonstrate that the present PA-Akt system enabled us to control spatiotemporal Akt activity and elucidate its functions in living cells.

### Computational modeling of the temporal dynamics of CRY2-Akt activity

**Figure 3:**
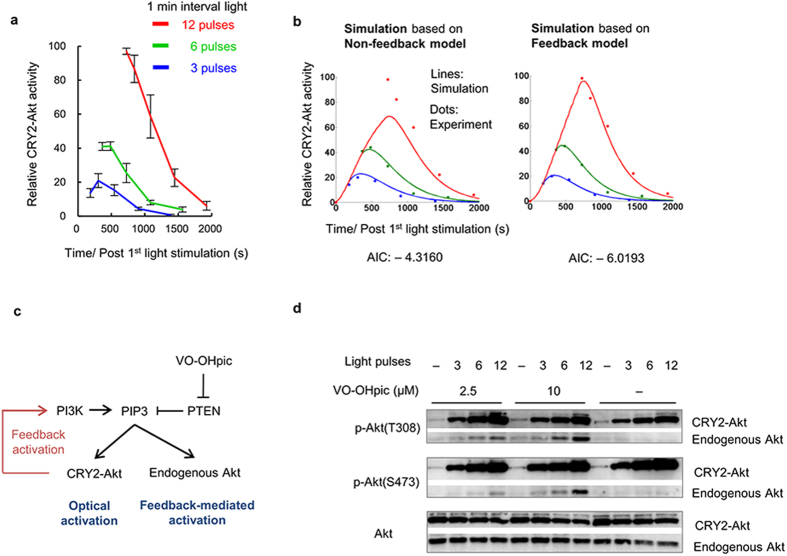
Computational modeling of temporal dynamics of CRY2-Akt activity. (**a**) Time courses of CRY2-Akt activity. C2C12 cells expressing Myr-CIBN and CRY2-Akt were stimulated 3, 6, or 12 times with light pulses at 1-min intervals. The relative CRY2-Akt activity was calculated using Western blot from Thr308 phosphorylation level of CRY2-Akt divided by the total amount of CRY2-Akt. The initiation of the first light pulse was set as time 0. Bars: Mean ± s.e.m. (*N* = 4, each in independent experiment). (**b**) Simulations of CRY2-Akt activation based on computational models of Non-feedback model (Supplementary Fig. 7) and Feedback model (Supplementary Fig. 8). The graph shows the time courses of CRY2-Akt activity in simulation (lines) and experiments (dots). A lower AIC value stands for a better fit. (**c**) Schematic of feedback-mediated PIP3 production by PI3K and the hydrolysis by PTEN. (**d**) Effects of PTEN inhibition on the activation of CRY2-Akt and endogenous Akt. C2C12 cells expressing Myr-CIBN and CRY2-Akt were pretreated with PTEN inhibitor, VO-OHpic for 15 min, and subsequently stimulated with 12 times of light pulses at 1-min interval.

To precisely control the temporal patterns of CRY2-Akt activity with light input, we developed computational models and estimated their parameters using experimentally obtained results of temporal CRY2-Akt patterns in cells stimulated with light pulses given at 1-min intervals under the optimized 4 mW/cm^2^ light intensity condition (Fig. 3a and Supplementary Fig. 6a). First, we constructed an abstract model (Non-feedback model) in which cytosolic inactive CRY2-Akt was translocated to the plasma membrane upon light stimulation and was subsequently activated by upstream molecules (Supplementary Fig. 7). However, the model with the set of estimated parameters was insufficient to predict the relative activation amplitude of CRY2-Akt, which was reproducible even under a different light intensity condition (1 mW/cm^2^) (Supplementary Fig. 6b). This result indicated that the nonlinear activation of CRY2-Akt was dependent on an unknown mechanism of Akt activation.

A positive feedback loop is a fundamental network structure in intracellular signaling including PI3K signaling that may account for the nonlinear activation of Akt^2^,^25^. Therefore, a feedback activation mode was incorporated into the model of Akt activation (Feedback model) (Supplementary Fig. 8). The results showed that the simulation of a positive feedback activation model reproduced both the temporal patterns and the amplitude of Akt activity (Fig. 3b). The model reproducibility was estimated using the Akaike Information Criterion (AIC)^26^, which is a general measure of the relative goodness of fitted models. The AIC of the Feedback model was smaller than that of the Non-feedback model (Fig. 3b), demonstrating that the fitting with the Feedback model was more reliable than the fitting with the Non-feedback model. Because AIC includes a penalty for the number of free parameters, the improved fitting is not simply derived from the increased parameters.

### Experimental validation of the developed computational model

To experimentally confirm the network structure of the positive feedback loop, endogenous Akt phosphorylation was examined using optical CRY2-Akt activation. The phosphorylation of endogenous Akt increased upon CRY2-Akt activation under a PTEN-phosphatase-inhibited condition (Fig. 3c,d). This endogenous Akt phosphorylation shows the elevation of PIP3 amount; the existence of the positive feedback loop from Akt to its upstream activator PI3K, of which the activity is attenuated with PTEN. Because CRY2phr-fused Akt does not contain its PH domain in the optimized construct, endogenous Akt activation implies the presence of elevated activity of upstream kinases of Akt, such as PDK1 and mTORC2; however increased endogenous Akt phosphorylation was also detected in cells expressing Myr-CIBN and CRY2phr fused with full-length Akt (Supplementary Fig. 9a). Fusion of the full-length Akt resulted in slightly higher activation amplitude upon stimulations of both light and insulin because the possession of PH domain increases membrane association probability (Supplementary Fig. 9a,b). The light-induced PIP3 production was confirmed using single-molecule imaging of a PIP3 reporter (tetramethylrhodamine (TMR)-labeled Akt) under a total internal reflection fluorescence microscope (TIRFM) (Supplementary Fig. 10a,b). The number of detected PIP3 reporters at the plasma membrane increased following CRY2-Akt activation, which was consistent with the increased endogenous Akt phosphorylation. To investigate the mechanism underlying the feedback loop, we tested the inhibition influence of several probable components of the feedback loop. Actin polymerization, which has been demonstrated to be perturbed by Akt (Supplementary Fig. 5a–c), is known to function as a driving force for cellular events including feedback loops^27^. The results showed that inhibition of actin polymerization with Latrunculin B (Lat.B) completely suppressed the positive feedback loop (Supplementary Fig. 11a). Suppression of endogenous Akt activity with Lat.B was also confirmed in cells stimulated with insulin (Supplementary Fig. 11b), as previously described in other cell lines^28^. Taken together, these data suggest that Akt is activated by a positive feedback loop mediated by PI3K activation and actin polymerization.

To qualitatively test the ability of the Feedback model to predict experimentally obtained results, we examined CRY2-Akt activity under genetic and pharmacological perturbations (Supplementary Fig. 12a). The PI3K inhibitors LY294002 and Wortmannin strongly attenuated the activation of CRY2-Akt (Supplementary Fig. 12b). These experimental features were reproduced by decreasing the rate constant of the parameter corresponding to PIP3 synthesis (k_6_ in Supplementary Fig. 7). Additionally, we investigated the effects of genetic perturbations on CRY2-Akt activity. The overexpression of wild-type PTEN attenuated the activation of CRY2-Akt (Supplementary Fig. 12c). In contrast, the expression of the dominant negative mutants, PTEN(C124S) and PTEN(R130Q), which are markers of tumorigenesis^29^, elevated the activation of CRY2-Akt (Supplementary Fig. 12d). These results were reproduced using simulations with the Feedback model. Taken together, these results suggest that the model with the estimated parameters correctly reconstituted the intracellular dynamics of CRY2-Akt.

**Figure 4:**
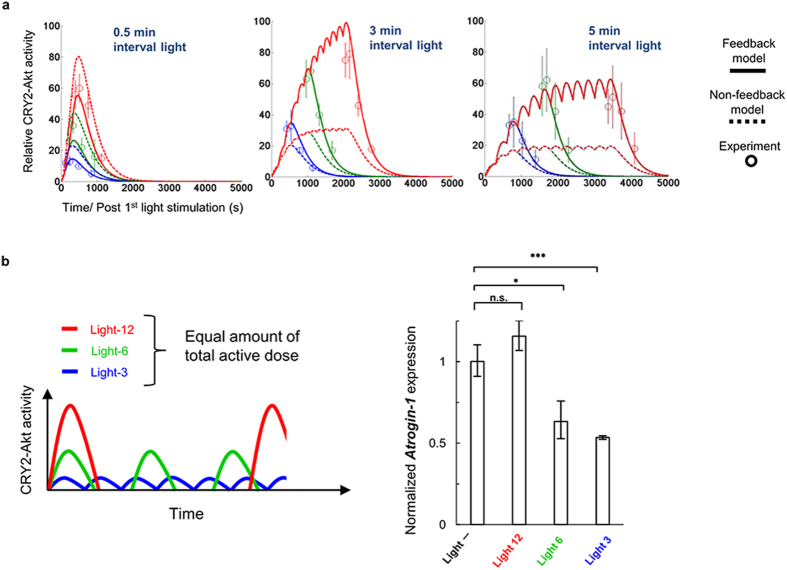
Predictive control of temporal patterns of CRY2-Akt activity with computational modeling. (**a**) Prediction of temporal patterns of CRY2-Akt activity in different intervals of light stimulation. C2C12 cells expressing Myr-CIBN and CRY2-Akt were stimulated with different intervals of light. Circles show the relative CRY2-Akt activity evaluated from the Thr308 phosphorylation divided by the total amount of CRY2-Akt. Lines and dotted lines respectively show simulations by the Feedback model and the Non-feedback model. Bars: Mean ± s.e.m. (*N* = 4, each in independent experiment). (**b**) Functional analysis of temporal Akt activity. Each of the CRY2-Akt activation pattern was generated by illuminating cells with 1-min interval of light pulses at 1 mW/cm^2^ intensity. *Atrogin-1* expression was measured at a time point of 200 min after the onset of light illumination. Activation interval: 16.0 min (Light-3), 36.5 min (Light-6), 91.0 min (Light-12). Last light pulse was added 6.0 min (Light-3), 12.5 min (Light-6), and 7.0 min (Light-12) before the collection of cells. *p < 0.05, ***p < 0.001 by a two-tailed Student’s *t*-test. n.s.: not significant. Bars: Mean ± s.e.m. (*N* = 3).

To quantitatively test the model’s ability to predict experimentally obtained results, we performed a cross-validation assay using new datasets of temporal CRY2-Akt patterns that were not used for the parameter estimations. We examined temporal CRY2-Akt patterns under different light stimulation conditions (0.5-min, 3-min, and 5-min intervals). The PA-Akt system generated different temporal patterns of CRY2-Akt activity under each light stimulation condition. These patterns were well predicted by the simulations based on the Feedback model, but not by the simulations based on the Non-feedback model (Fig. 4a). Specifically, simulations based on the Feedback model predicted a lower activation amplitude of CRY2-Akt under the 0.5-min interval time condition better than the simulations based on the Non-feedback model, indicating that CRY2-Akt was activated with feedback-mediated increasing speed. Consequently, we concluded that the Feedback model enabled the predictive control of temporal patterns of CRY2-Akt activity even under different light stimulation conditions and genetic and pharmacological perturbations.

### Functional analysis of temporal Akt dynamics

Finally, to demonstrate the utility of the developed system with the computational model, we investigated cellular responses in cells with different temporal patterns of Akt activity. The cells were stimulated with three different patterns of Akt activity (Light-12, Light-6, and Light-3) as an input using custom-built LED arrays (Fig. 4b, left). *Atrogin-1* expression was measured as a cellular output. The total active CRY2-Akt dose was set to an equal amount between different stimulation patterns based on the mathematical model simulations. Intriguingly, *Atrogin-1* expression decreased under Light-3 and Light-6 conditions but not Light-12, which produced a higher amplitude and lower frequency of Akt activity (Fig. 4b, right). This result strongly suggests that cells have a mechanism by which specific temporal patterns of Akt activity are captured as an informative cellular input.

## Discussion

Here, we introduced an optogenetic system and a computational mode to precisely control the temporal dynamics of Akt activity. Although a chemically inducible dimerization system that can control Akt activity has previously been described^30^, the present approach has several advantages: the reversibility of the system on a timescale of minutes, the ability to control activity spatially, functionality without exogenous cofactor addition, and the predictability of the light-induced Akt activity output. These characteristics enabled us to reconstitute physiological Akt dynamics, whose functional significance has been suggested but not investigated. Although the predictability of the light-induced output is limited to temporal dynamics at present, the strategy of light-induced output prediction with computational modeling can be extended to spatial dynamics. Furthermore, the overall strategy is applicable to other optogenetic systems, thereby offering a general framework for manipulating optogenetic tools in a precise quantitative manner.

Although the approach with the model-based predictive control described here would provide an important advance in the optogenetic control of Akt, there are several limitations at present. First, the model is not directly applicable to the CRY2-Akt dynamics induced by other stimulations, such as extracellular growth factors, because our model currently assumes only the contribution of light to CRY2-Akt dynamics. For instance, insulin stimulation activates several signaling pathways simultaneously in addition to Akt (Fig. 1c), which probably affects the temporal dynamics of CRY2-Akt. For the same reason, the precision of model prediction may sometimes be inaccurate when the system is used in a condition where other factors that perturb CRY2-Akt dynamics exist, such as under an *in vivo* condition. Second, the simulation did not fully match the experimental data when the cells were illuminated with intervals of light other than 1-min. This discrepancy may originate from errors in the experimental data used for parameter estimations owing to the difficulty of precisely quantifying phosphorylated Akt in western blotting assays. The development of more precise quantification methods will strengthen the present system in terms of model prediction.

In an attempt to construct a computational model of temporal Akt dynamics, we found that Akt used a positive feedback activation mode mediated by PI3K activation and actin polymerization. In accordance with previous studies^23^,^24^,^31^,^32^, we found that optical activation of Akt was sufficient to perturb cellular polarity, thereby supporting the existence of the positive feedback activation mode. Feedback networks play a key role in generating spatially and temporally confined signals to break symmetry^2^,^25^. We anticipate that the detailed mechanism of the actin-related feedback mechanism will be revealed by taking advantage of the developed optogenetic system to perturb Akt activity at a single cell level.

In summary, we developed an optogenetic system that enabled specific regulation of Akt activity in a spatiotemporal manner within its physiological dynamic range, and demonstrated optical control of the downstream functions of Akt. Furthermore, the system achieved the precise control of Akt activity using a computational model, thereby providing direct evidence that the temporal patterns of Akt activation are pivotal in inducing its biological outcome. The model with experimental data indicated that Akt was activated by a positive feedback loop through PI3K activation and actin polymerization. Owing to the central roles of Akt in various biological processes and human diseases, the ability to precisely control its activity at the desired level is expected to facilitate versatile applications especially in therapeutic treatment and synthetic biology. The present approach of optogenetic output prediction with computational modeling will provide a general strategy that can be used to interrogate the functional significance of the temporal dynamics of biomolecules.

## Methods

### Reagents

Specific antibodies for pAkt(Thr308)(#2965), pAkt(Ser473)(#4051), Akt(#4691), pGSK3α/β(S21/9)(#9331), GSK3(#5676), p-ERK1/2(T202/Y204)(#4370), ERK1/2(#4695), and PTEN(#9559) were obtained from Cell Signaling Technology (MA). VO-OHpic and Wortmannin were obtained from Sigma Aldrich. Insulin, LatrunculinB, and LY294002 were obtained from Wako Pure Chemical Industries (Japan).

### Cell culture and retrovirus infection

C2C12, HEK293, and PlatE cells were maintained in DMEM supplemented with 10% FBS, 100 units/ml penicillin and 100 μg/ml streptomycin at 37 °C in 5% CO_2_. For retrovirus infection of C2C12 cells, plasmid DNA was transfected with the *Trans*IT-LT1 reagent (Takara, Japan) into packaging cells (PlatE cells). After 2 days of culture, high-titer retroviruses were collected and used to infect C2C12 cells. To achieve high expression of the optical modules, multiple rounds of infection were conducted at 6-h intervals. Cells were seeded onto 35-mm dishes for western blot assays and onto 24-well plates for quantitative RT-PCR assays.

### Construction of plasmids

The cDNAs encoding human Akt1/PKBα (original ORF clone: ORK02215; Kazusa, Japan), human PTEN (original ORF clone: FXC09870; Kazusa, Japan), CRY2 and CIB1 (Addgene, MA) were amplified by a standard PCR with gene specific primers. The membrane-targeting myristoylation and palmitoylation signal sequence (MGCVQCKDKEATKLTE) originated from the sequence of Fyn. The respective efficiencies of membrane translocation were almost equal for full-length CRY2 and CRY2PHR (amino acids 1–498) (Supplementary Fig. 1b) as described previously^18^. Point mutations were introduced by mutagenic complement oligo single-stranded DNA pairs. These constructs were inserted into pcDNA vectors (Invitrogen, CA) for transient expression in mammalian cells or the pMX vector for retrovirus infection.

### Western blotting

Prior to the assays, the culture medium of C2C12 cells in 35-mm dishes was replaced with phenol red-free DMEM supplemented with 0.1% FBS and carefully maintained in the dark overnight at 37 °C in 5% CO_2_. The serum-starved cells were stimulated with light pulses using a blue-LED transilluminator (470 nm, LEDB-SBOXH; Optocode Corp., Japan), then lysed with 200 μL of sampling buffer (5% SDS, 10% glycerol, 10% 2-mercaptoethanol, and 125 mM Tris-HCl, pH 6.8) and gently sonicated for 3–5 min. Insoluble debris was removed by centrifugation at 15,000 × *g* at 4 °C for 15 min. The sample was boiled at 95 °C for 5 min and separated on SDS-polyacrylamide gels. The electrophoretically-separated proteins were transferred onto PVDF membranes (GE Healthcare, UK) and, blocked with 1% skimmed-milk in Tris-buffered saline containing Tween-20 (TBS-T: 150 mM NaCl, 0.05% Tween-20, and 50 mM Tris-HCl, pH 8.0) for at least 1 h, then subsequently immunoblotted with specific antibodies. The immunostained bands were detected using ECL detection reagents with an image analyzer (LAS-1000; Fuji Film, Japan). The intensity of the immunoblotted bands was measured using the ImageJ software. It was possible to activate the present system with sustained light illumination. However, a pulse light illumination protocol was adopted in this study to rapidly activate CRY2-Akt with minimal effects due to chromophore bleaching and photo-toxicity.

### Quantitative RT-PCR

For RT-PCR, cells in each well of 24-well plates were stimulated using a custom LED array controlled by a microcontroller (Arduino UNO). LEDs (470 nm, 60° illumination, OptoSupply, China) were placed underneath the wells of a 24-well plate to stimulate the cells in each well with an independent illumination protocol at an intensity of 1 mW/cm^2^. Although 4 mW/cm^2^ intensity of light achieved the fastest activation of CRY2-Akt (Supplementary Fig. 3), this condition yielded large variability in the expression level of the internal control gene *cyclophilin A* (particularly at the 270 min time point) probably because of the cytotoxicity of light stimulation. Therefore, we adopted a light intensity of 1 mW/cm^2^ in the qRT-PCR assays. At the 1 mW/cm^2^ intensity, the expression level of the internal control gene was relatively stable throughout the experiment. The light-illuminated cells were fixed with 300 μL of RNAlater (Qiagen, Netherlands), and total RNA was isolated using the Agencourt RNAdvance Tissue kit (Beckman Coulter, CA). The isolated RNAs were reverse transcribed to cDNAs by the PrimeScript RT reagent Kit (Takara, Japan) and measured by real-time quantitative RT-PCR with gene-specific primers (*Atrogin-1*: forward [5′-CAGCTTCGTGAGCGACCTC-3′] and reverse [5′-GGCAGTCGAGAAGTCCAGTC-3′], *cyclophilin A*: forward [5′- GAGCTGTTTGCAGACAAAGTTC-3′] and reverse [5′- CCCTGGCACATGAATCCTGG-3′]). We calculated the relative abundance of *Atrogin-1* mRNA using *cyclophilin A* mRNA as an internal control. PCR reactions were performed using SYBR Premix Ex TaqII (Takara, Japan) in a Thermal Cycler Dice Real-time system TP-800 (Takara, Japan).

### Time-lapse imaging

In the assays to evaluate the light-dependent translocation of CRY2-Akt, HEK293 cells or C2C12 cells were seeded onto a 35-mm glass-bottom dish in phenol red-free DMEM supplemented with 10% FBS, 100 unit/mL penicillin and 100 μg/mL streptomycin. After 24 h of incubation at 37 °C in 5% CO_2_, the cells were transfected with plasmid DNAs. Two days after transfection, the cells were observed using a confocal fluorescence microscope (FV1000-D; Olympus Corp., Japan) with a PlanApo 60× oil immersion objective (N.A.: 1.35), 515-nm laser for Venus excitation, and 559-nm laser for mCherry and RFP excitation. The illumination power for the photo-activation of CRY2-Akt was 0.5–3.0%, with a 20 mW 440-nm laser light at a speed of 2.0 μs/pixel. For the FoxO1-mCherry translocation assays, C2C12 cells expressing Myr-CIBN and CRY2-Akt were seeded onto a 35-mm glass-bottom dish. Then, the cells were transfected with a plasmid encoding FoxO1-mCherry after 24 h of incubation. After an additional 24 h of incubation, the cells were starved with phenol red-free DMEM supplemented with 0.1% FBS and carefully maintained in the dark overnight at 37 °C in 5% CO_2_. Activation of CRY2-Akt was conducted with 1-min intervals of 440-nm laser light 6–12 times in the middle of each experiment. The expression of Myr-CIBN and CRY2-Akt was confirmed to not perturb CRY2-Akt activity at the end of the assays via exposure to the excitation light for fluorescent proteins. The fluorescence intensities of the cytosol and nucleus were quantified using the ImageJ software. The N/C ratio change was calculated by dividing an average N/C ratio after light stimulation by the ratio prior to light stimulation. The 440-nm wavelength of laser light used for photo-activation in the time-lapse imaging assays differed from the 470-nm wavelength of LED light used for the Western blot assays and qRT-PCR assays because of the limitation of the microscopic setup. Because the absorption spectrum of the photoreceptor CRY2 was broad (ranging from 400−500 nm), both wavelengths of light were functional in activating the optogenetic system. In the present stage, it was not problematic to activate the system with two light sources that had different activation efficiencies because we did not directly compare the data from single cells to the data from cell populations.

### Quantification of PIP3 production

Cells expressing Akt-SNAPf were treated with 10 nM TMR-conjugated SNAPf ligands for 20 minutes and washed three times with PBS. The cells were serum-starved overnight in HBSS containing 0.1% FBS and subjected to the PIP3 quantification assay. Single molecule imaging of Akt-SNAPf(TMR) was performed using an inverted microscope (IX-81; Olympus Corp., Japan) equipped with a home-built TIRF apparatus, a 561 nm laser and a PlanApo 100×oil immersion objective (N.A.: 1.45). Fluorescence signals from TMR were detected with a cooled EM-CCD camera (ImagEM; Hamamatsu photonics, Japan). Aquacosmos and HC-Image software (Hamamatsu photonics, Japan) were used to control the microscope system and capture the fluorescence images, respectively. The images were taken at a speed of 30 frames per second, and a total of 100 frame images were analyzed. Light illumination was conducted using a blue-LED apparatus that generated a 2 mW/cm^2^ intensity of 470-nm light at the sample point. The obtained images were analyzed with the ImageJ plugin Particle Tracker. The number of detected molecules was normalized to the images collected prior to stimulations.

### Quantification of cell polarity

C2C12 cells expressing the optogenetic module and Lifeact-RFP were stimulated with single 440-nm laser pulses under a confocal microscope. To calculate the polarity index (cos*θ*) of the migrating C2C12 cells, the x and y coordinates were obtained for the centroid before movement, the centroid after movement, and for the center of the irradiation spot by analyzing the Lifeact-RFP image using the ImageJ software.

### Computational modeling of Akt activation

We developed a light-dependent Akt activation model based on the law of mass action and performed simulation and parameter estimations using Matlab (R2012b) and the Systems Biology Toolbox 2 (SBTOOLBOX2) for MATLAB. The parameters in the model were estimated using experimental data from Fig. 3a according to two methods in series: First, a meta-evolutionary programming method was used to approach the neighborhood of the local minimum. Second, the Nelder–Mead method was used to reach the local minimum^33^. After 200-independent estimations for the model, we selected the model that had the minimum value for the objective function, which was defined as the residual sum of the squares between the experimentally obtained results and the simulations.
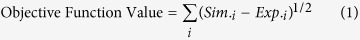


Note that the pathways and molecules in the model do not directly correspond to the real biochemical pathway because of the abstract model. We estimated the model parameters from experimentally obtained results of Thr308 of CRY2-Akt, because the time course of Ser473 was nearly the same as that of Thr308 (Supplementary Fig. 6a). A model comparison was conducted using the AIC value from each model. The AIC value was defined as follows.
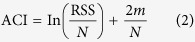
Therein, *N* denotes the number of data points, *m* denotes the number of estimating parameters, and RSS denotes the residual sum of squares.

### Statistical analysis

Data are presented as the mean ± s.e.m. unless otherwise mentioned. Statistical significance was determined using unpaired or paired student’s *t*-tests (two-tailed). *P* values < 0.05 were considered statistically significant.

## Additional Information

**How to cite this article**: Katsura, Y. *et al.* An optogenetic system for interrogating the temporal dynamics of Akt. *Sci. Rep.* **5**, 14589; doi: 10.1038/srep14589 (2015).

## Supplementary Material

Supplementary Information

Supplementary Video 1

Supplementary Video 2

Supplementary Video 3

Supplementary Video 4

Supplementary Video 5
