# Supplementary material for: An optogenetic system for interrogating the temporal dynamics of Akt
Source: Sci Rep. 2015 Oct 1;5:14589. doi: 10.1038/srep14589 (PMC4589684; doi:10.1038/srep14589)
Supplement: Supplementary Information [file srep14589-s1.pdf]

Supplementary Information

An optogenetic system for interrogating the temporal dynamics of Akt

Yoshihiro Katsura[a], Hiroyuki Kubota[b, c, d], Katsuyuki Kunida[b, d], Akira Kanno[a], Shinya Kuroda[b, d], Takeaki Ozawa[a, d, *],

a. Department of Chemistry, School of Science, The University of Tokyo, 7-3-1 Bunkyo-ku, Hongo, Tokyo 113-0033, Japan

b. Department of Biophysics and Biochemistry, School of Science, The University of Tokyo, 7-3-1 Bunkyo-ku, Hongo, Tokyo 113-0033, Japan

c. Division of integrated Omics, Research Center for Transomics Medicine, Medical Institute of Bioregulation, Kyushu University, 3-1-1, Maidashi, Higashi-ku, Fukuoka, Fukuoka 812-8582, Japan

d. CREST, Japan Science and Technology Agency, 4-1-8 Honcho, Kawaguchi, Saitama, 332-0012, Japan

* Correspondence should be addressed to T.O.

E-mail: ozawa@chem.s.u-tokyo.ac.jp

Tel.: 81-3-5841-4351

Fax: 81-3-5802-2989

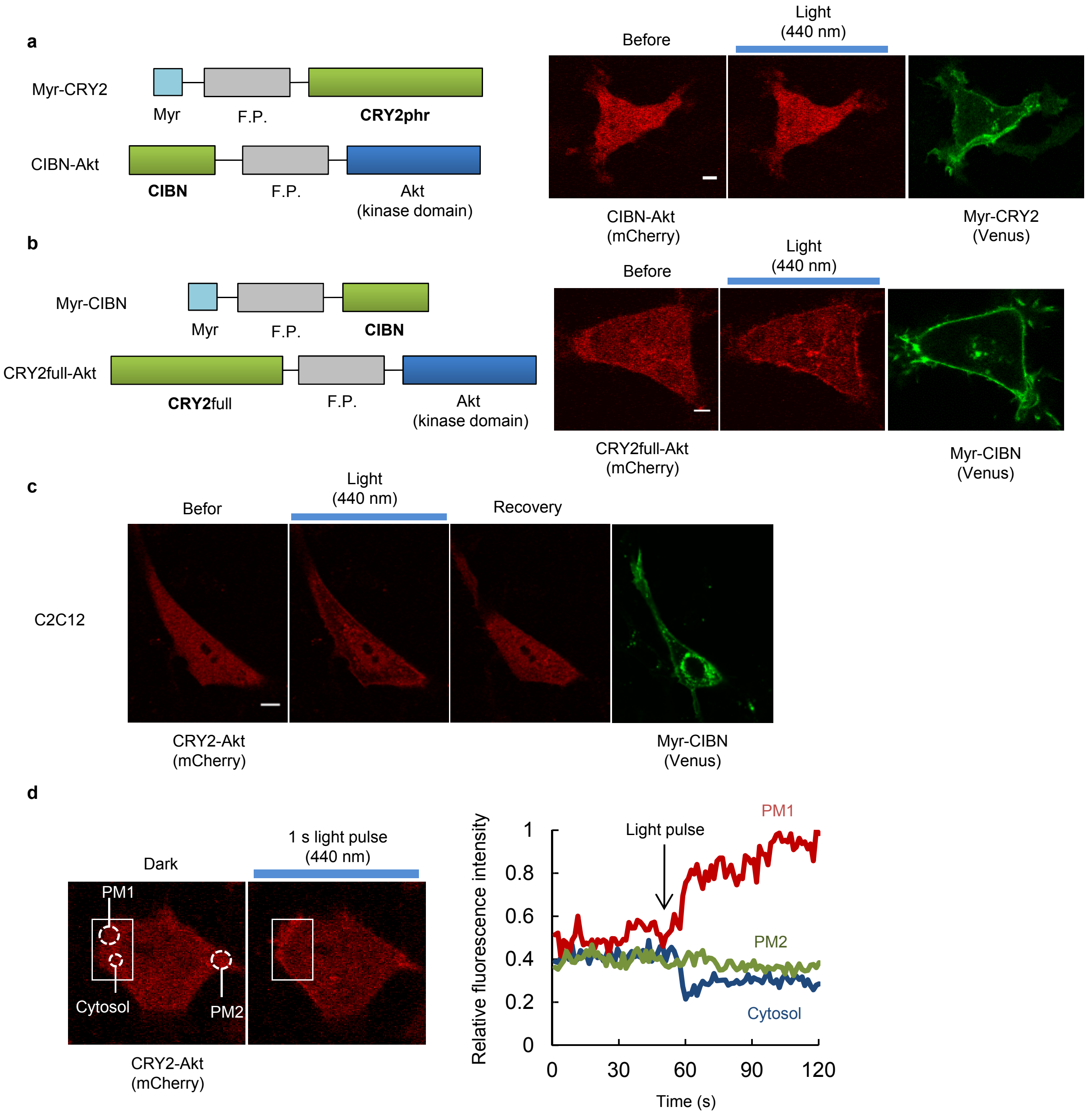

Supplementary Figure 1: Single-cell level characterization of a PA-Akt system.
(a) CIBN-Akt was not recruited to the cell membrane upon light in the cells expressing Myr-CRY2 and CIBN-Akt. CRY2 was localized at the plasma membrane by fusing the membrane targeting myristoylation sequence. Green: Myr-CRY2, Red: CIBN-Akt. Scale bar, 10 µm. **(b)** Full-length CRY2 ($CRY2_{full}$) was used as a dimerization domain with CIBN. The photoreaction kinetics is approximately the same as a PHR domain of CRY2. Green: Myr-CIBN, Red: $CRY2_{full}$-Akt. Scale bar, 10 µm. **(c)** Photo-response of CRY2-Akt in C2C12 cell. Green: Myr-CIBN, Red: CRY2-Akt. Scale bar, 10 µm. **(d)** Subcellular control of CRY2-Akt localization in the same cell as shown in **Fig. 1b**. Square region was stimulated with 440-nm laser light for 1 s. Graph shows the time courses of normalized fluorescence intensity per pixel at three different regions in the cell. PM1: Plasma membrane at light-stimulated region. PM2: Plasma membrane at stimulated region. Cytosol: cytosol within the light stimulated area.

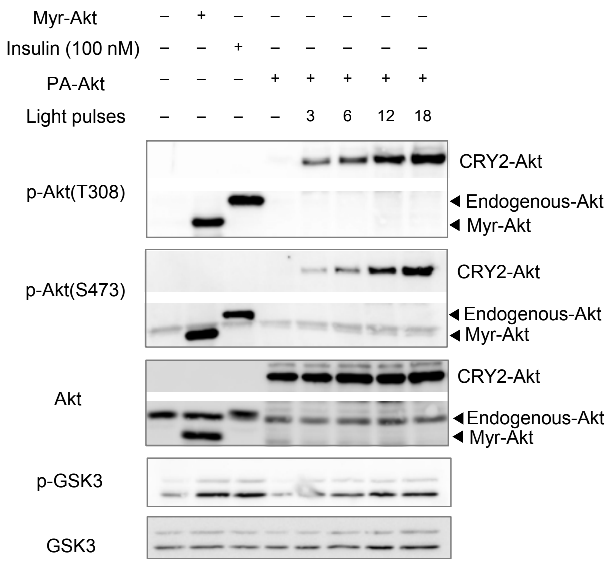

Supplementary Figure 2: **Comparison with a constitutively active Akt (Myr-Akt).**
CRY2-Akt was activated in a comparable level to a constitutively membrane-localized Akt (Myr-Akt) and endogenous Akt. Endogenous Akt was full-activated with 100 nM insulin for 10 min.

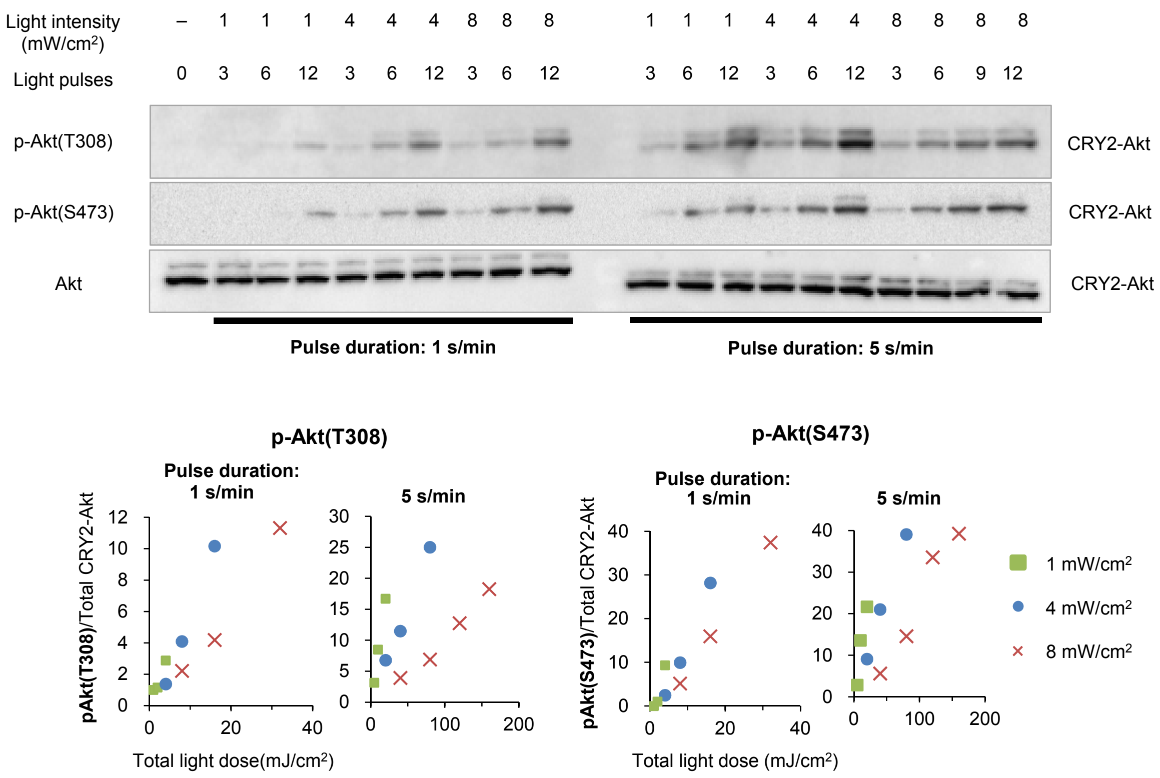

Supplementary Figure 3: Characterization of intensity and duration of light pulses.
Light-induced activation of CRY2-Akt was evaluated in different light intensity, duration, and the times of pulses. Serum-starved C2C12 cells expressing Myr-CIBN and CRY2-Akt were stimulated with light pulses. The cells were collected 1 min after final light pulse and subjected to Western blot analysis. Activation intensity was approximately proportional to the times of light pulses in all light conditions. The maximum activation was achieved in the cells stimulated with 4 mW/cm^2 intensity for 5 s duration per minute. Graphs show the relative CRY2-Akt activity calculated from the immunoblotting band intensity. The total light dose is defined as a multiplicative result of intensity, duration, and times of light pulses.

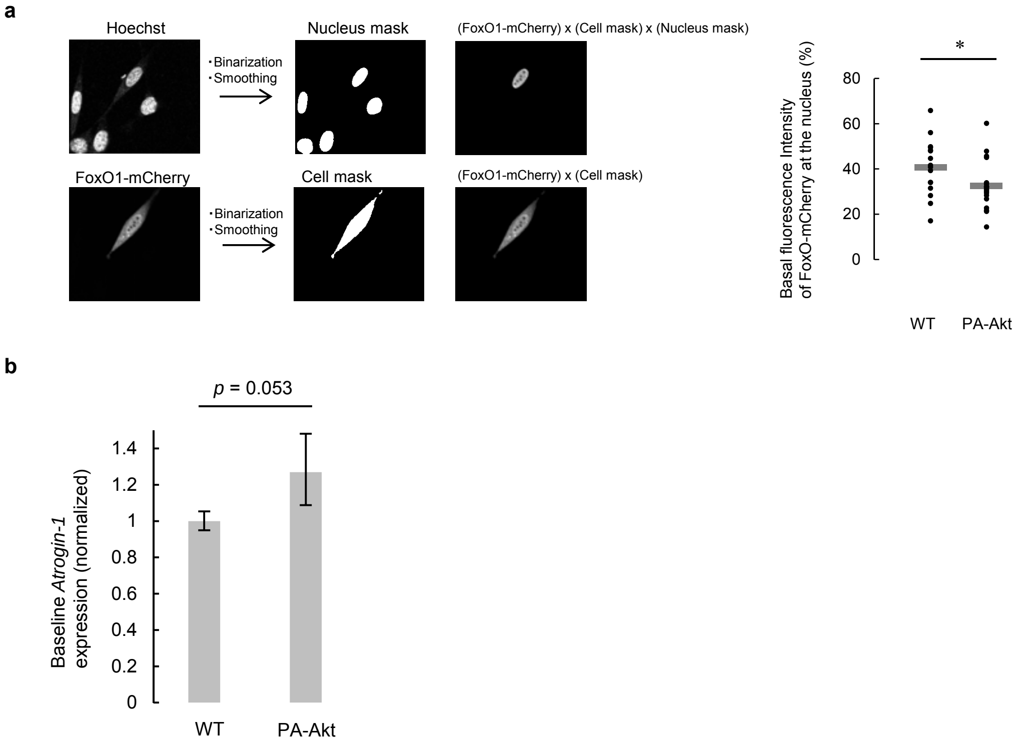

Supplementary Figure 4: **Baseline differences induced by the expression of Myr-CIBN and CRY2-Akt.** **(a)** Basal activity of Myr-CIBN and CRY2-Akt to FoxO1-mCherry localization. The degree of nuclear localization of FoxO1-mCherry in the dark was quantified by using the cell and nucleus mask (*N*=15 for wild type C2C12 cells and *N*=19 for C2C12 cells expressing Myr-CIBN and CRY2-Akt). Cells were stained with a nuclear marker, Hoechst33342, and subsequently fixed with formaldehyde. **(b)** Basal effect of Myr-CIBN and CRY2-Akt to *Atrogin-1* expression. *Atrogin-1* expression was measured with quantitative RT-PCR assays (*N*=4 for each condition).

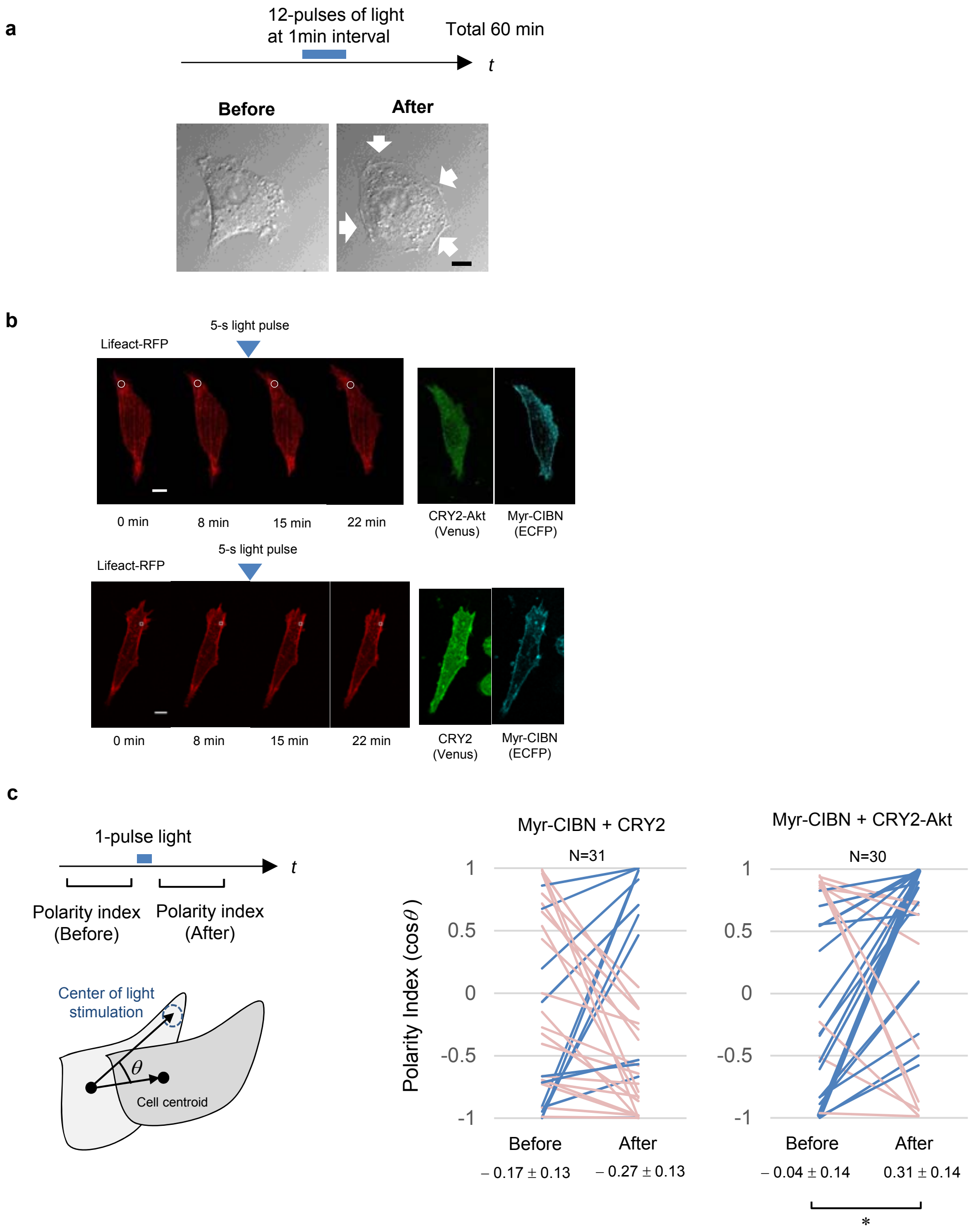

Supplementary Figure 5: Optical control of Akt-induced actin remodeling.
(a) Membrane ruffling formation induced by CRY2-Akt activation with 440-nm laser light stimulation. Arrows indicate the formation of membrane ruffling. Before image, 5 min before the first light pulse; After image, 13 min after the final light pulse; Scale bar, 10 µm. **(b)** Representative images of light-induced cell migration. Scale bar, 10 µm. Light-activation spot diameter, 5µm. **(c)** Statistical analysis of a light-induced cell migration by a focal Akt activation. Red lines indicate cells migrating to the light-illuminated spot while the blue lines indicate the cells migrating opposite direction from the light-illuminated spot (N=31 for control cells expressing Myr-CIBN and CRY2, N=30 for cells expressing Myr-CIBN and CRY2-Akt, * p<0.05 by a two-tailed paired t-test).

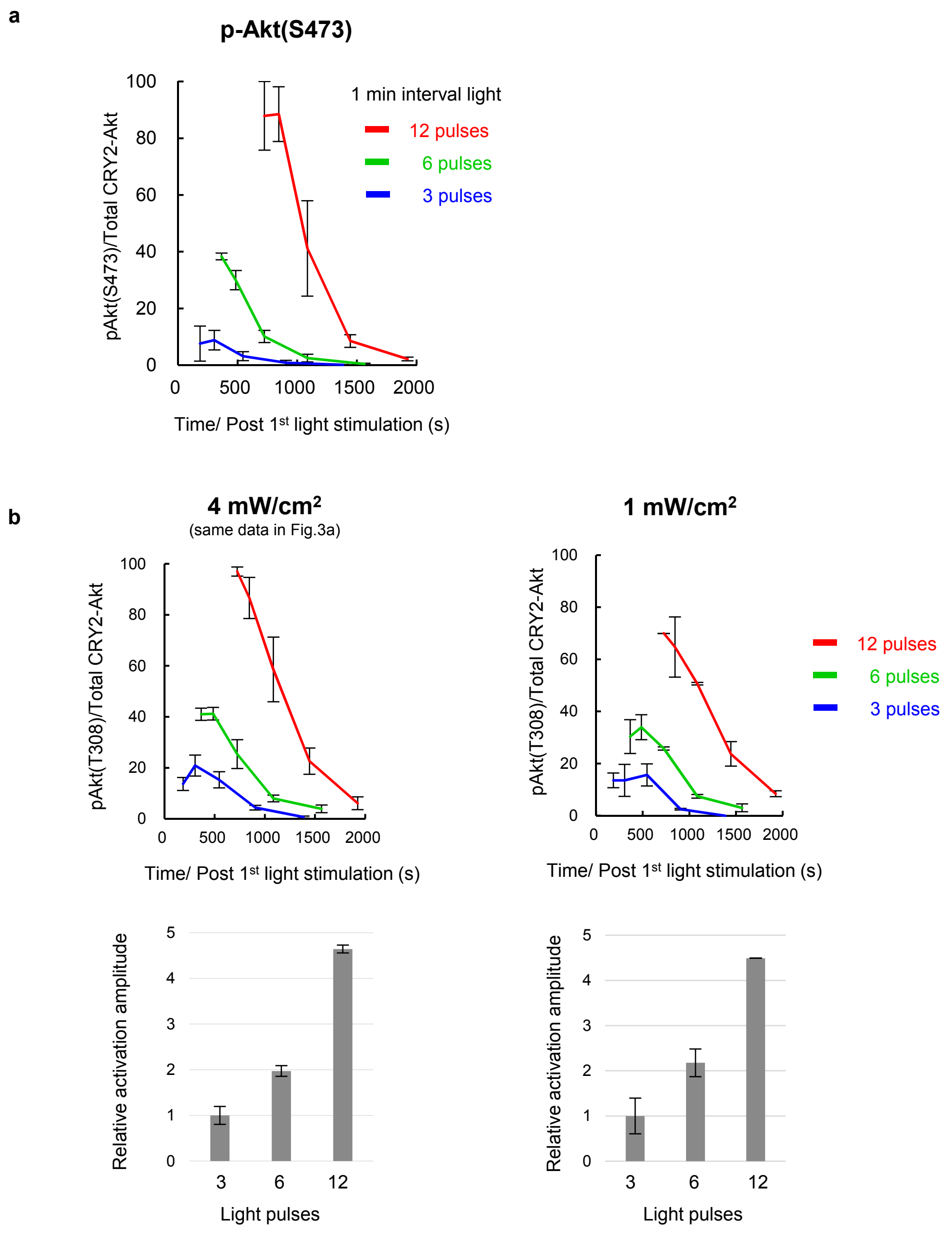

Supplementary Figure 6: Characterization of CRY2-Akt activity.
(a) Time courses of Ser473 phosphorylation in CRY2-Akt. C2C12 cells expressing Myr-CIBN and CRY2-Akt were stimulated with different numbers of light pulses at 1-min interval. Time course of Thr308 phosphorylation was shown as a reference. Bars: Mean ± s.e.m. (*N*=4, each in independent experiments). **(b)** Dependency of light intensity in the relative activation amplitude of CRY2-Akt. Almost the same relative activation amplitude was detected between 1 mW/cm^2 and 4 mW/cm^2 intensity of light. In the upper time-course graphs, each CRY2-Akt amplitude was shown in relative to the amplitude of 1 min post 12 light pulses at 4 mW/cm^2 intensity condition. In the lower bar graphs, the relative activation amplitude between different numbers of light pulses was calculated from the highest CRY2-Akt activity in each time course. Bars: Mean ± s.e.m. (*N*=4, each in independent experiment).

a

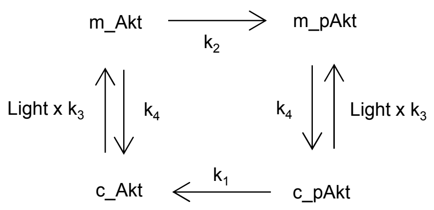

k_5 = Total Akt
Light = 0 or 1
Total pAkt = m_pAkt + c_pAkt

b

	Reaction	Reaction constant (/sec)	PN
A	c_pAkt → c_Akt	$2.7903x10^{-3}$	k1
B	mAkt → m_pAkt	$4.7152x10^{-3}$	k2
C	m_pAkt ⇄ c_pAkt	$5.3363x10^{-3}$, $3.5866x10^{-3}$	k3,k4
D	m_Akt ⇄ c_Akt	$5.3363x10^{-3}$, $3.5866x10^{-3}$	k3,k4

c Initial amounts

Molecular name	Initial amounts	Parameter
c_pAkt	0	–
m_pAkt	0	–
c_Akt	$1.0000x10^{3}$	k5
m_Akt	0	–

d ODEs

Differential equation	Re
$\frac{d[c\_pAkt]}{dt}$ = - k1•[c_pAkt]	A
+ Light•k3•[m_pAkt]	C
- k4•[c_pAkt]	C
$\frac{d[m\_pAkt]}{dt}$ = k2•[m_Akt]	B
- Light•k3•[m_pAkt]	C
+ k4•[c_pAkt]	C
$\frac{d[c\_Akt]}{dt}$ = k1•[c_pAkt]	A
- Light•k3•[c_Akt]	D
+ k4•[m_Akt]	D
$\frac{d[m\_Akt]}{dt}$ = - k2•[m_Akt]	B
+Light•k3•[c_Akt]	D
- k4•[m_Akt]	D

Supplementary Figure 7: Computational model of non-feedback CRY2-Akt activation.
(a) Schematic of the non-feedback model: m_Akt, non-phosphorylated CRY2-Akt at membrane; c_Akt, non-phosphorylated CRY2-Akt at cytosol; m_pAkt, phosphorylated CRY2-Akt at membrane; c_pAkt, phosphorylated CRY2-Akt at cytosol. **(b)** Reactions and rate constants in the model. **(c)** Initial amounts of molecules in the model. **(d)** Ordinary differential equations (ODEs) in the model. Simulations were performed using Matlab (See also Methods).

a

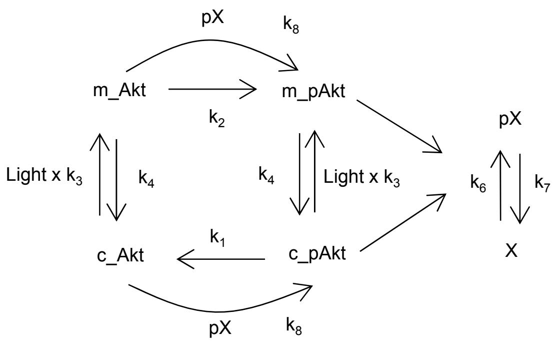

k_5 = Total Akt
k_9 = Total X
Light = 0 or 1
Total pAkt = m_pAkt + c_pAkt

b

	Reaction	Reaction constant (/sec)	Parameter
A	c_pAkt → c_Akt	3.1300×10^{-5}	k1
B	mAkt → m_pAkt	3.4503×10^{-3}	k2
C	m_pAkt ⇄ c_pAkt	4.8355×10^{1}, 7.0148×10^{-3}	k3,k4
D	m_Akt ⇄ c_Akt	4.8355×10^{1}, 7.0148×10^{-3}	k3,k4
E	pX ⇄ X	4.6710×10^{-2}, 5.5892×10^{-1}	k6,k7
F	m_Akt + pX → m_pAkt + pX	4.1519×10^{-4}	k8
G	c_Akt + pX → c_pAkt + pX	4.1519×10^{-4}	k8

c Initial amounts

Molecular name	Initial amounts	Parameter
c_pAkt	0	–
m_pAkt	0	–
c_Akt	8.9873×10^{2}	k5
m_Akt	0	–
pX	0	–
X	6.7211×10^{-1}	k9

d ODEs

Differential equation	Reaction
$\frac{d[c\_pAkt]}{dt} = - k1 \cdot [c\_pAkt]$	A
$- Light \cdot k3 \cdot [m\_pAkt]$	C
$+ k4 \cdot [c\_pAkt]$	C
$+ k8 \cdot [c\_Akt] \cdot [pX]$	G
$\frac{d[m\_pAkt]}{dt} = k2 \cdot [m\_Akt]$	B
$+ Light \cdot k3 \cdot [m\_pAkt]$	C
$- k4 \cdot [c\_pAkt]$	C
$+ k8 \cdot [m\_Akt] \cdot [pX]$	F
$\frac{d[c\_Akt]}{dt} = k1 \cdot [c\_pAkt]$	A
$- Light \cdot k3 \cdot [c\_Akt]$	D
$+ k4 \cdot [m\_Akt]$	D
$\frac{d[m\_Akt]}{dt} = - k2 \cdot [m\_Akt]$	B
$+ Light \cdot k3 \cdot [c\_Akt]$	D
$- k4 \cdot [m\_Akt]$	D
$\frac{d[pX]}{dt} = k6 \cdot [X] \cdot ([m\_pAkt]+[c\_pAkt])$	F, G
$- k7 \cdot [pX]$	E

Supplementary Figure 8: Computational model of feedback-mediated CRY2-Akt activation.
(a) Schematic of the Feedback model: m_Akt, non-phosphorylated CRY2-Akt at membrane; c_Akt, non-phosphorylated CRY2-Akt at cytosol; m_pAkt, phosphorylated CRY2-Akt at membrane; c_pAkt, phosphorylated CRY2-Akt at cytosol. X; Feedback-comprising molecule in inactive state. pX; Feedback-comprising molecule in active state. **(b)** Reactions and rate constants in the model. **(c)** Initial amounts of molecules in the model. **(d)** Ordinary differential equations (ODEs) in the model. Simulations were performed using Matlab (See also Methods).

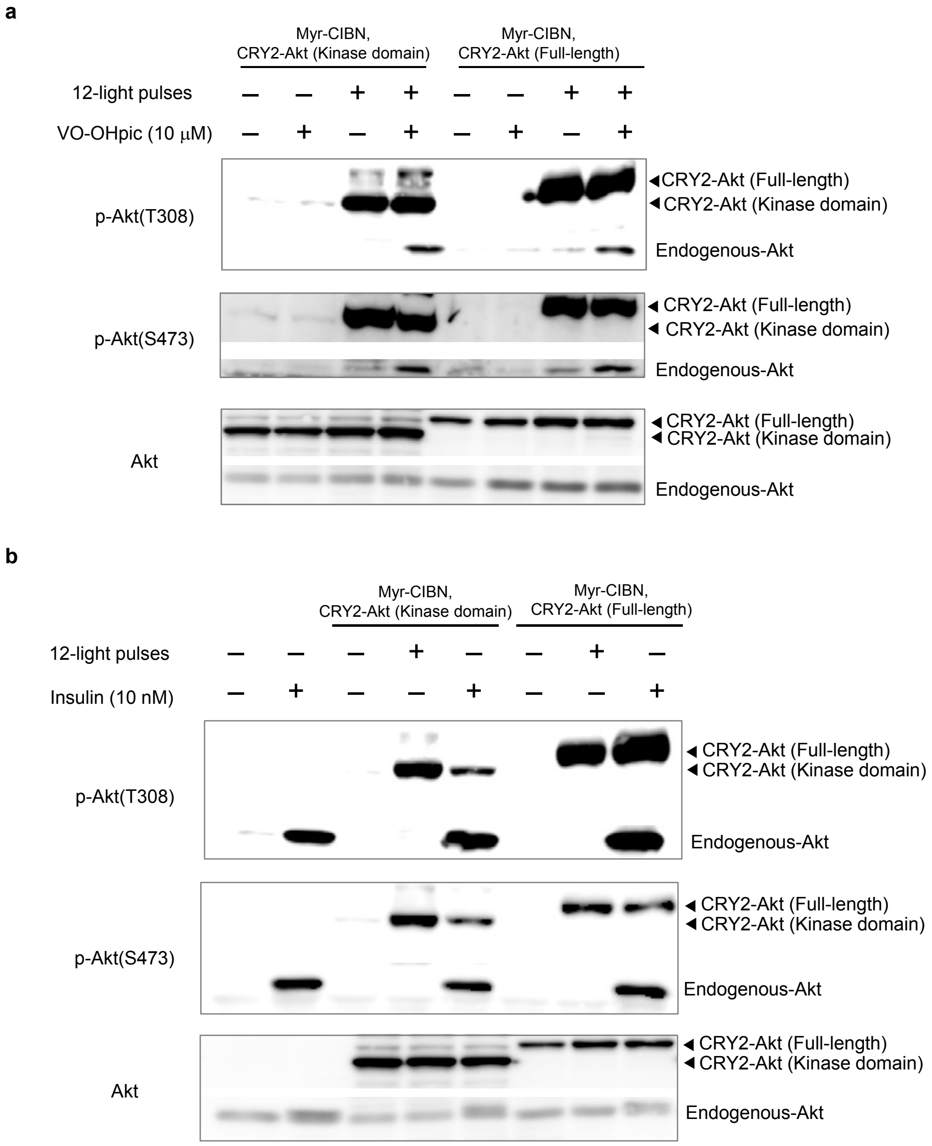

Supplementary Figure 9: Characterization of a CRY2phr fused with a full-length of Akt.
(a) Related to **Fig. 3d**, phosphorylation of endogenous Akt was examined upon activation of CRY2-Akt(full-length). C2C12 cells expressing Myr-CIBN and CRY2-Akt(kinase domain) or CRY2-Akt(full-length) were pretreated with PTEN inhibitor, VO-Ohpic for 15 min, and subsequently stimulated with 12 times of light pulses at 1-min interval. **(b)** Activation of CRY2-Akt with light or insulin. Cells were treated with 12-light pulses at 1-min interval or with 10 nM insulin for 10 min. The degree of T308 phosphorylation with insulin was weaker in CRY2-Akt(kinase domain) than in CRY2-Akt(full-length), enabling specific activation with light illumination.

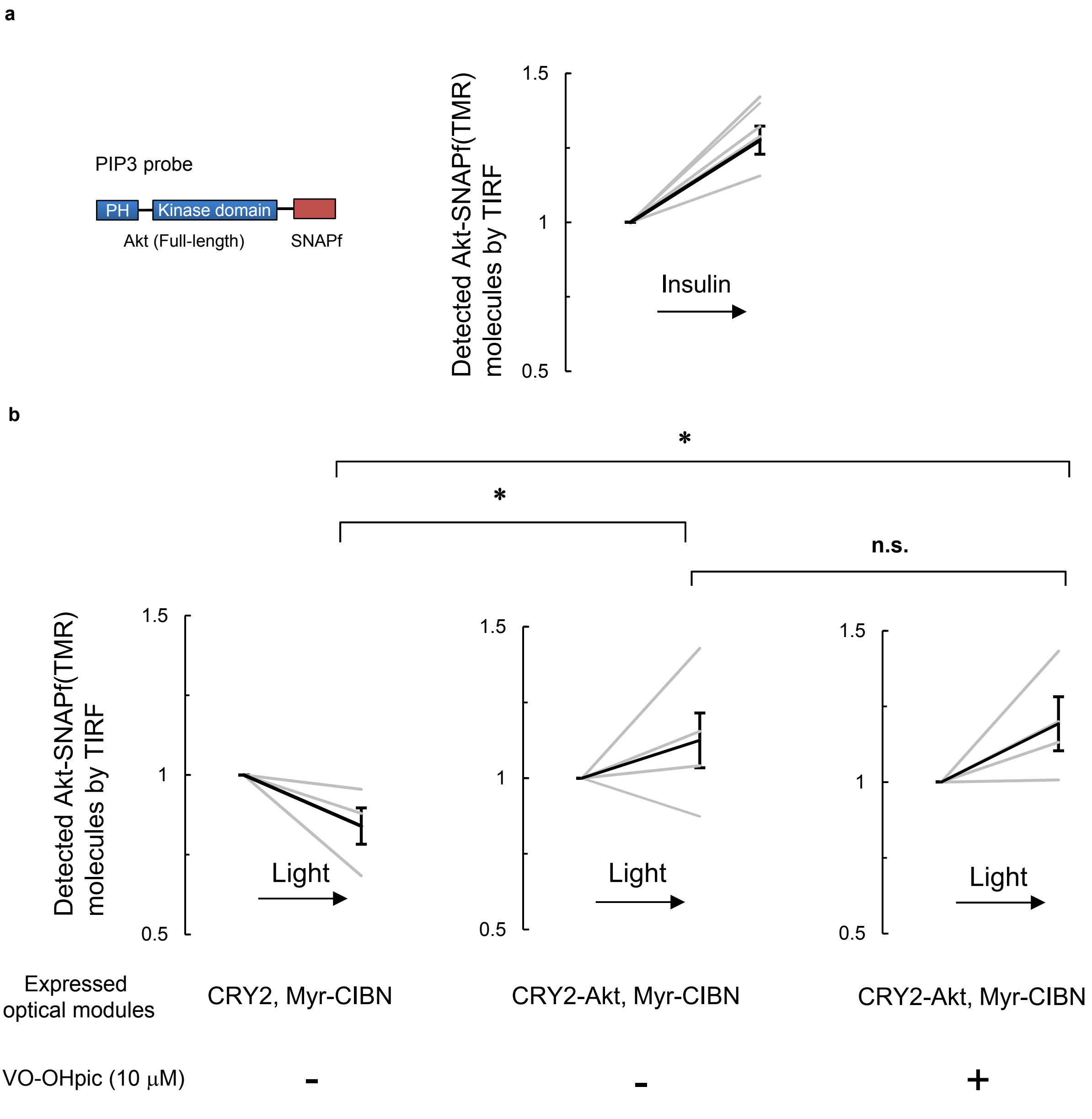

Supplementary Figure 10: PIP3 production induced by CRY2-Akt activation.
(a) A PIP3 reporter responsible for insulin stimulation. C2C12 cells expressing Akt-SNAPf conjugated with tetramethylrhodamine (Akt-SNAPf(TMR)) were stimulated with 10 nM insulin for 15 min. Single molecules of Akt-SNAPf(TMR) in the plasma membrane were detected using TIRF microscope. Bars: Mean ± s.e.m. (*N*=6). **(b)** C2C12 cells expressing Myr-CIBN, Akt-SNAPf(TMR), and CRY2 or CRY2-Akt were stimulated with 12 times of light pulses at 1-min interval. * $p<0.05$ by a two-tailed Student's *t*-test. n.s.: not significant. Bars: Mean ± s.e.m. (*N*=5 for each experimental condition).

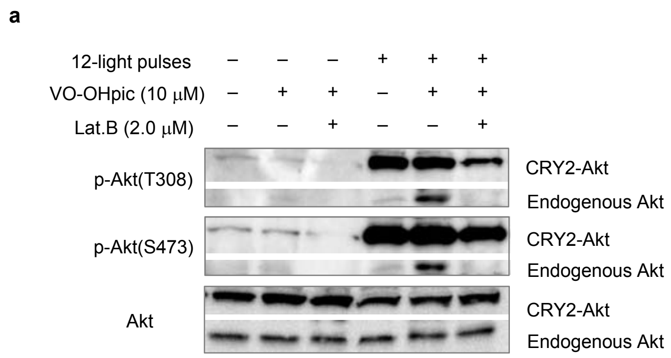

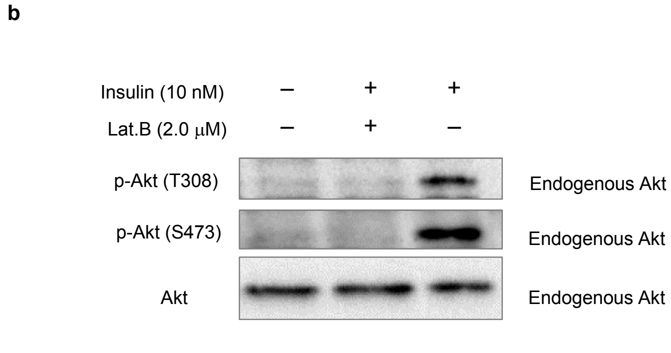

Supplementary Figure 11: Elucidation of the activation mechanism of Akt.
(a) Inhibition of the positive feedback loop by an actin polymerization inhibitor, LatrunculinB (Lat.B). Cells expressing Myr-CIBN and CRY2-Akt were pretreated with 2 μM Lat.B and 10 μM VO-OHpic for 15 min, and were subsequently stimulated 12 times at 1-min intervals with light pulses. **(b)** Inhibition of insulin-induced endogenous Akt activation by Lat.B. Serum-starved C2C12 cells were treated with insulin (10 nM) and Lat.B (2.0 μM) for 15 min and subjected to Western blot.

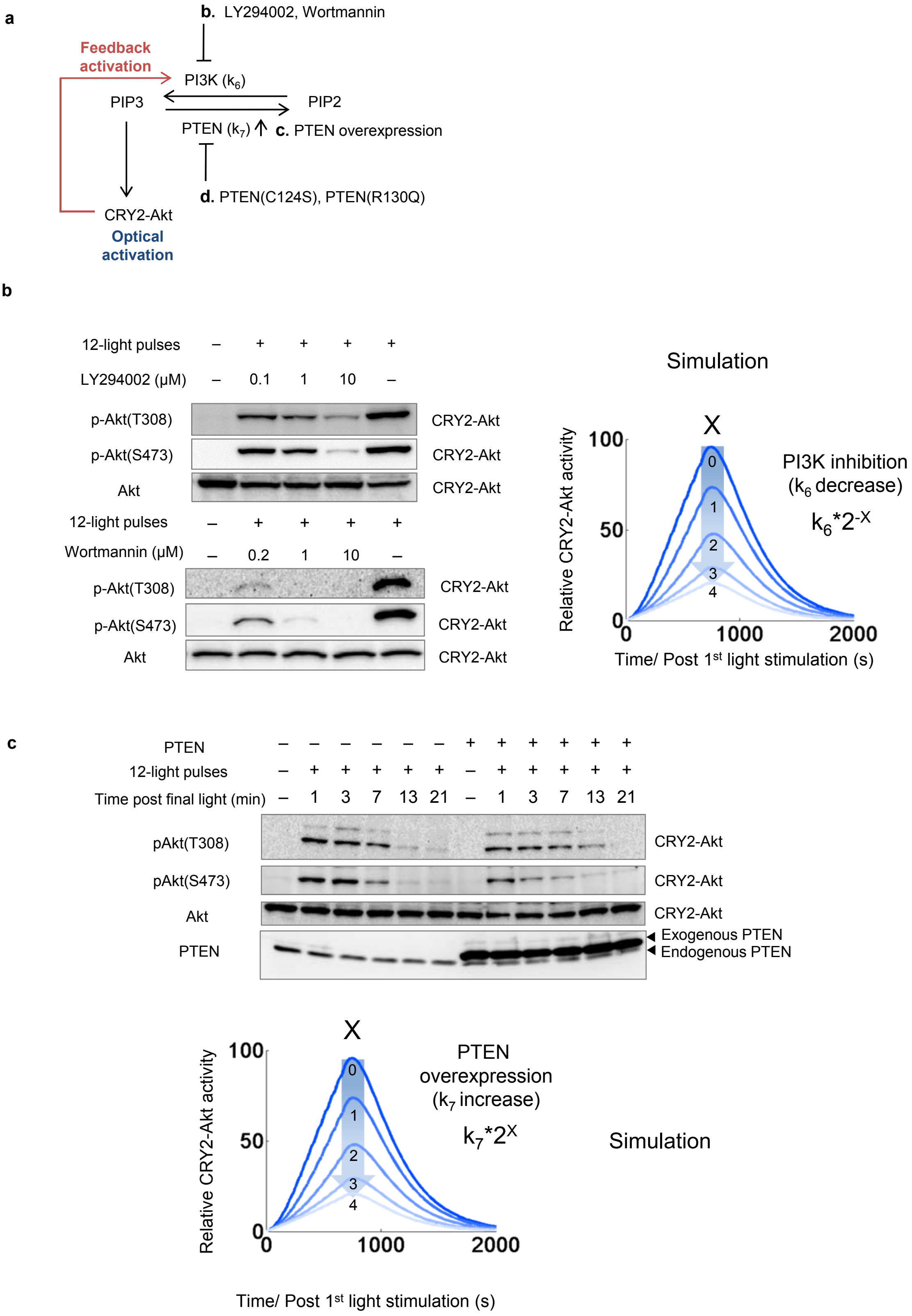
a
b. LY294002, Wortmannin
Feedback activation
PI3K (k6)
PIP3
PIP2
PTEN (k7)
c. PTEN overexpression
d. PTEN(C124S), PTEN(R130Q)
CRY2-Akt
Optical activation
b
12-light pulses – + + + +
LY294002 (μM) – 0.1 1 10 –
p-Akt(T308)
p-Akt(S473)
Akt
CRY2-Akt
12-light pulses – + + + +
Wortmannin (μM) – 0.2 1 10 –
Simulation
X
PI3K inhibition
(k6 decrease)
k6*2-X
Relative CRY2-Akt activity
Time/ Post 1st light stimulation (s)
c
PTEN – – – – – – + + + + + +
12-light pulses – + + + + + – + + + + +
Time post final light (min) – 1 3 7 13 21 – 1 3 7 13 21
pAkt(T308)
pAkt(S473)
Akt
PTEN
CRY2-Akt
Exogenous PTEN
Endogenous PTEN
PTEN overexpression
(k7 increase)
k7*2X
Simulation
Relative CRY2-Akt activity
Time/ Post 1st light stimulation (s)

d

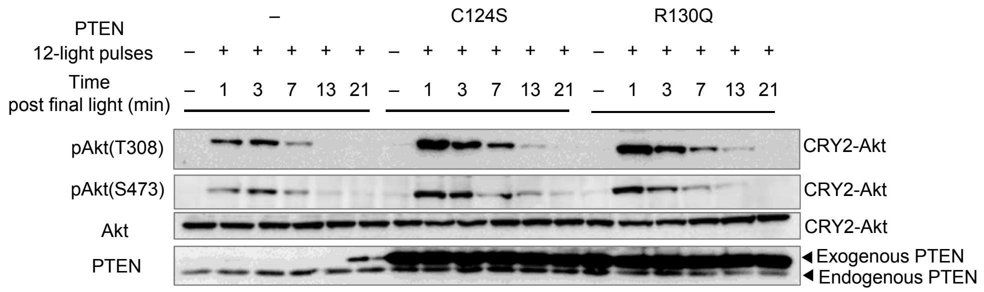

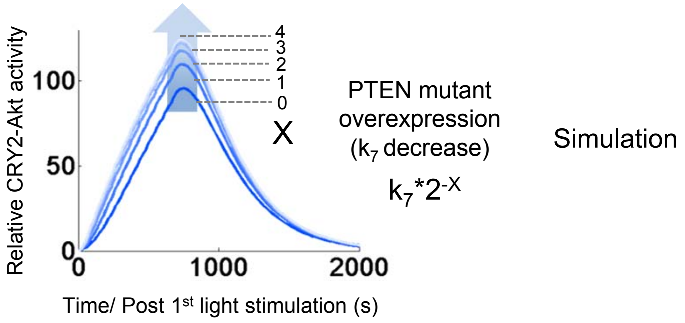

Supplementary Figure 12: Validation of the developed computational model structure with genetic or pharmacological perturbations.

(a) Schematic of genetic and pharmacological perturbations on CRY2-Akt. **(b)** (Left) Effect of PI3K inhibition on the activation of CRY2-Akt. C2C12 cells expressing Myr-CIBN and CRY2-Akt were pretreated with LY294002 (upper) or Wortmannin (lower) for 10 min and were subsequently stimulated 12 times with light pulses. (Right) Simulations of PI3K inhibitor effect on CRY2-Akt activity. The amplitude of CRY2-Akt activation decreased by decreasing the rate constant of the parameter corresponding to PIP3 synthesis (k_6 in **Supplementary Fig. 8**). The initiation of the first light pulse was set as time 0. **(c)** (Upper) Effect of PTEN overexpression on the CRY2-Akt activity. C2C12 cells expressing Myr-CIBN, CRY2-Akt, and wild-type PTEN were stimulated 12 times with light pulses. (Lower) Simulations of the effects of PTEN expression on the CRY2-Akt activity. The amplitude of CRY2-Akt activation decreased by increasing the rate constant of the parameter corresponding to PIP3 hydrolysis (k_7 in **Supplementary Fig. 8**). **(d)** (Upper) Effect of PTEN mutant expression on the CRY2-Akt activity. C2C12 cells expressing Myr-CIBN, CRY2-Akt and dominant negative PTEN mutants (PTEN(C124S), PTEN(R130Q)) were stimulated 12 times with light pulses. (Lower) Simulations of the effects of PTEN mutant expression on the CRY2-Akt activity. The amplitude of CRY2-Akt activation increased by decreasing the rate constant of the parameter corresponding to PIP3 hydrolysis (k_7 in **Supplementary Fig. 8**).

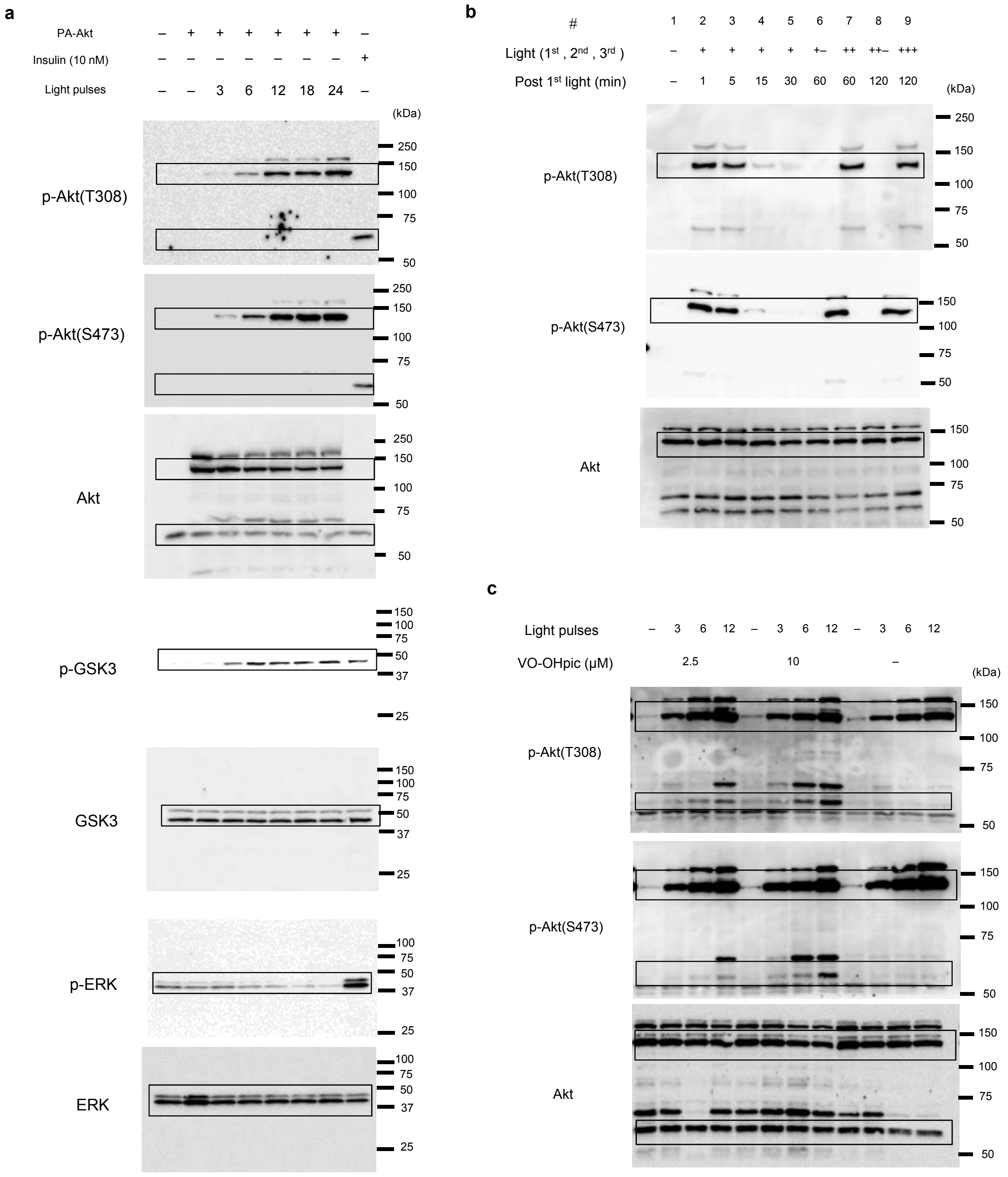
a
PA-Akt
Insulin (10 nM)
Light pulses
3 6 12 18 24
(kDa)
p-Akt(T308)
p-Akt(S473)
Akt
p-GSK3
GSK3
p-ERK
ERK
b
#
Light (1st , 2nd , 3rd)
Post 1st light (min)
1 5 15 30 60 60 120 120
p-Akt(T308)
p-Akt(S473)
Akt
c
Light pulses
VO-OHpic (μM)
2.5
10
p-Akt(T308)
p-Akt(S473)
Akt

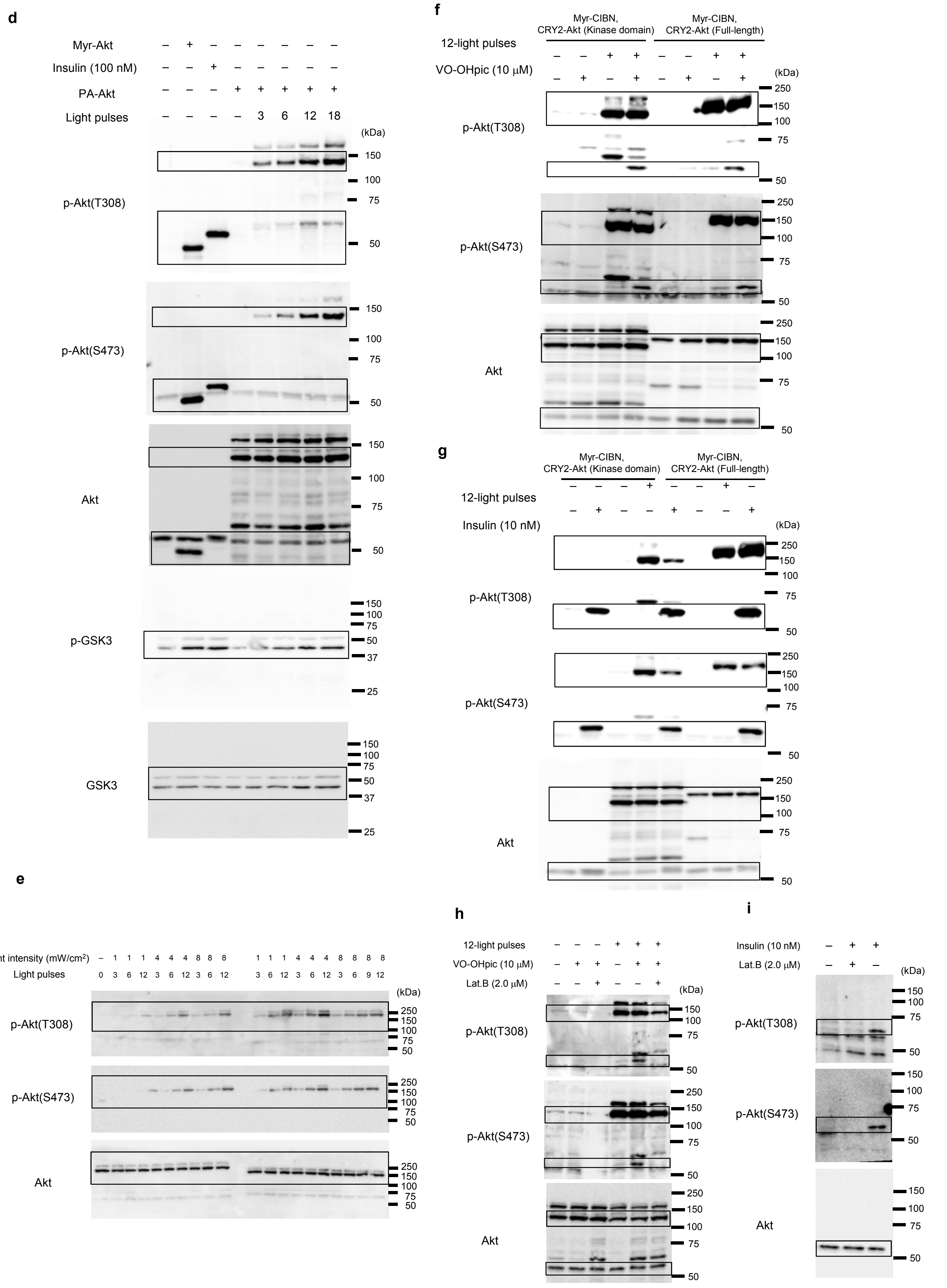
d
Myr-Akt
Insulin (100 nM)
PA-Akt
Light pulses
3 6 12 18
(kDa)
p-Akt(T308)
p-Akt(S473)
Akt
p-GSK3
GSK3
e
Light intensity (mW/cm2)
Light pulses
p-Akt(T308)
p-Akt(S473)
Akt
f
Myr-CIBN, CRY2-Akt (Kinase domain)
Myr-CIBN, CRY2-Akt (Full-length)
12-light pulses
VO-OHpic (10 μM)
p-Akt(T308)
p-Akt(S473)
Akt
g
Myr-CIBN, CRY2-Akt (Kinase domain)
Myr-CIBN, CRY2-Akt (Full-length)
12-light pulses
Insulin (10 nM)
p-Akt(T308)
p-Akt(S473)
Akt
h
12-light pulses
VO-OHpic (10 μM)
Lat.B (2.0 μM)
p-Akt(T308)
p-Akt(S473)
Akt
i
Insulin (10 nM)
Lat.B (2.0 μM)
p-Akt(T308)
p-Akt(S473)
Akt

j

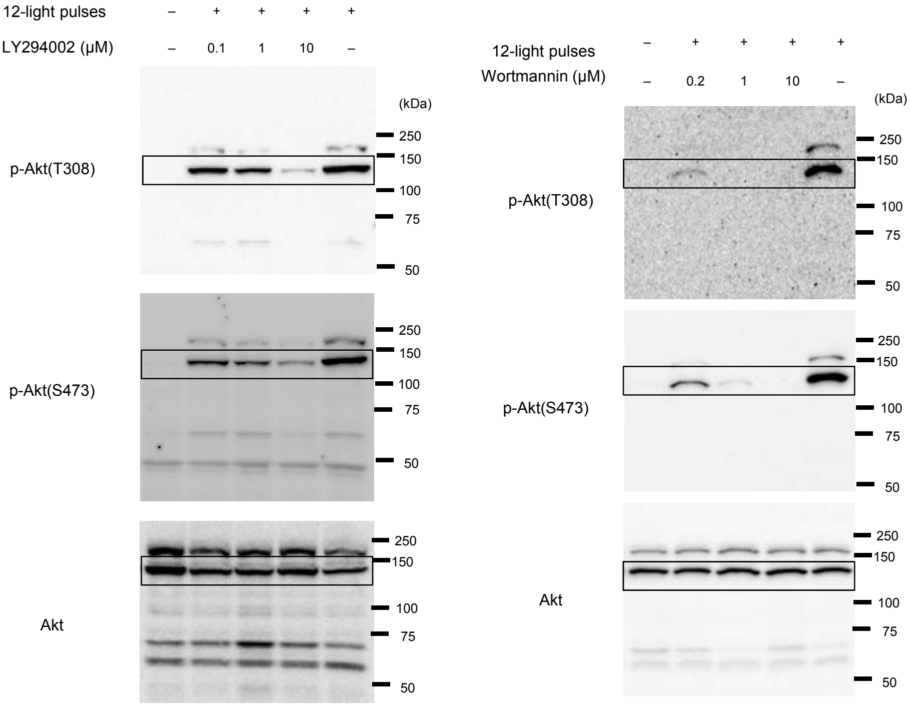

k

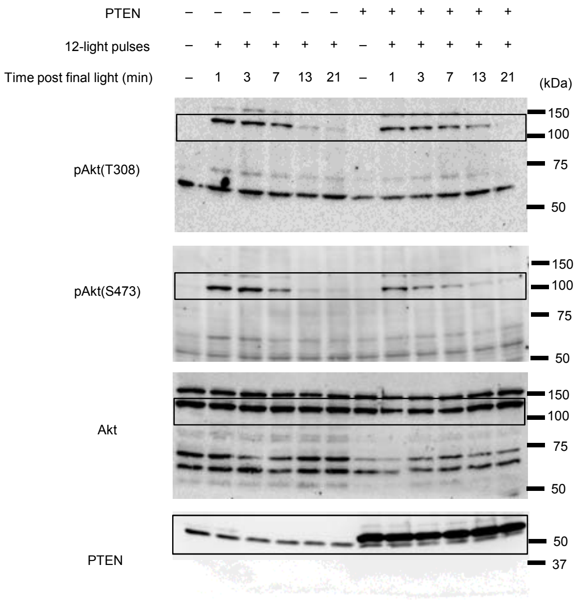

l

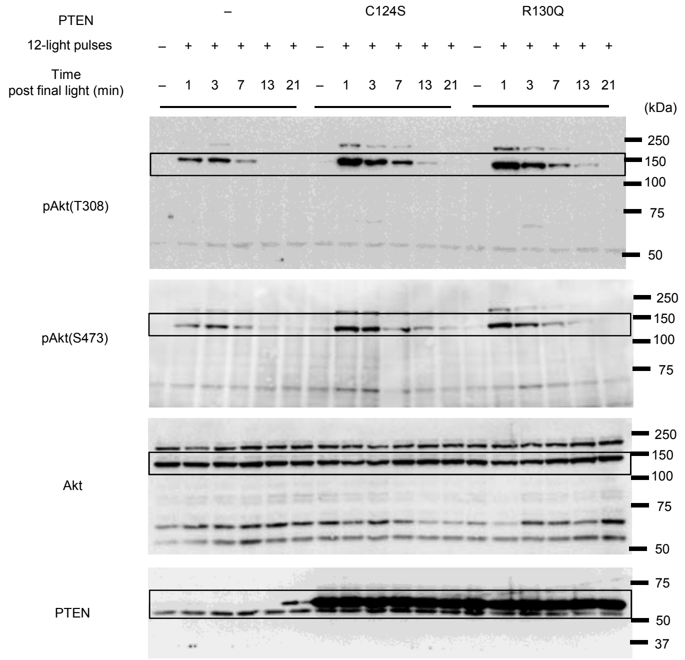

Supplementary Figure 13: Full blots images.
(a) Full blots images of **Fig. 1c**. **(b)** Full blots images of **Fig. 1d**. **(c)** Full blots images of **Fig. 3d**. **(d)** Full blots images of **Supplementary Fig. 2**. **(e)** Full blots images of **Supplementary Fig. 3**. **(f)** Full blots images of **Supplementary Fig. 9a**. **(g)** Full blots images of **Supplementary Fig. 9b**. **(h)** Full blots images of **Supplementary Fig. 11a**. **(i)** Full blots images of **Supplementary Fig. 11b**. **(j)** Full blots images of **Supplementary Fig. 12b**. **(k)** Full blots images of **Supplementary Fig. 12c**. **(l)** Full blots images of **Supplementary Fig. 12d**.

Video

Video 1: Photo-response of CRY2-Akt with a global light stimulation.

HEK 293 cell expressing Myr-CIBN and CRY2-Akt was stimulated with 440-nm laser light for 5 s. Scale bar, 5 μm.

Video 2: Photo-response of CRY2-Akt with a focal light stimulation.

In HEK 293 cell expressing Myr-CIBN and CRY2-Akt, square region was stimulated with 440-nm laser light for 1 s.

Video 3: Translocation of FoxO1-mCherry upon CRY2-Akt activation.

Serum-starved C2C12 cell expressing Myr-CIBN, CRY2-Akt and FoxO1-mCherry was stimulated with 440-nm laser light. Oscillatory translocation of FoxO1 between nucleus and cytoplasm was observed. Scale bar, 10 μm.

Video 4: Formation of membrane ruffling upon CYR2-Akt activation.

Serum-starved C2C12 cell expressing Myr-CIBN and CRY2-Akt was stimulated with 440-nm laser light 12 times at 1-min interval. Scale bar, 10 μm.

Video 5: Cell migration induced by a focal light stimulation.

Serum-starved C2C12 cell expressing Myr-CIBN, CRY2-Akt and Lifeact-RFP was stimulated with single 440-nm laser light pulse. Scale bar, 10 μm. ROI spot diameter, 5 μm.
